# The complete chloroplast genome of *Cryptomeria japonica* var*. sinensis* (Cupressaceae)

**DOI:** 10.1080/23802359.2020.1821812

**Published:** 2020-09-21

**Authors:** Wei-Wei Xie, Jun-Nan Li, Bao-Jian Ye, Fei-Ping Zhang

**Affiliations:** a Forestry College, Key Laboratory of Integrated Pest Management in Ecological Forests, Fujian Province University, Fujian Agriculture and Forestry University, Fuzhou, P. R. China; b College of Architecture and Urban Planning, Fujian Universtiy Of Technology, Fuzhou, P. R. China

**Keywords:** *Cryptomeria japonica* var. *Sinensis* Miquel, chloroplast genome, Cupressaceae, phylogenomic analysis

## Abstract

The complete chloroplast genome of *Cryptomeria japonica* var. *sinensis* Miquel was assembled and analyzed. The chloroplast genome of *C. japonica* var. *sinensis* Miquel did not have a typical quadripartite structure with the inverted repeats (IR) absent, and the size of *C. japonica var. sinensis* is 131,412 bp. The overall GC content was 35.4%. The genome encoded a set of 119 genes, containing 83 protein-coding genes, 32 tRNA genes, and 4 rRNA genes. Phylogenomic analysis indicated that *C. japonica* var. *sinensis* is sister to *C. japonica* (Thunb. ex L. f.) D. Don.

*Cryptomeria japonica* var. *sinensis* Miquel is a variant of the *C. japonica* (Thunb. ex L. f.) D. Don. It is beautiful in tree figure, capable of purifying the air and improving the environment, which makes it one of the primary afforestation tree species in high altitude regions of south China and an important Garden ornamental tree species (Dou et al. 2010). Currently, its natural forests are mainly distributed in Wuyi Mountain in Fujian and Tianmu Mountain in Zhejiang across southeast China, and broadly introduced at different provinces in China. Consequently, it has great prospects and the genetic resources are worth preservation (Wang et al. 2007). According to the Flora of China, there are obvious morphological differences between *C. japonica* var. *sinensis* Miquel and *C. japonica* (Thunb. ex L. f.) D. Don. However, whether their chloroplast genomes differed remains unclear. In this study, the completed chloroplast genome sequence of *C. japonica* var. *sinensis* Miquel is reported contributing for better understanding its evolution and population genetics, and also providing significant information for the phylogeny of Cupressaceae.

The fresh leaves of *C. japonica* var. *sinensis* Miquel were collected from Fujian province, China (Xiapu, Ningde: 119°56′37′′E, 26°52′19′′N). Voucher specimen (specimen code FJFC8003) was deposited in the Key Laboratory of Integrated Pest Management in Ecological Forests, Fujian Province University, Fujian Agriculture and Forestry University. Total genomic DNA was extracted from 100 mg fresh leaves using a modified CTAB method (Murray and Thompson 1980). Libraries with an average length of 350 bp were constructed using the NexteraXT DNA Library Preparation Kit. Sequencing was performed on the Illumina Novaseq 6000 platform (Total Genomics Solution Limited, SZHT), and the average length of the generated reads was 150 bp. The Illumina raw sequence reads were edited using the NGS QC Tool Kit version 2.3.3(National Institute of Plant Genome Research (NIPGR), New Delhi, India).High-quality reads were assembled into contigs using the *de novo* assembler SPAdes version 3.11.0 (Bankevich et al. 2012) and finally annotated by PGA software (Qu et al. 2019).

Like some gymnosperms chloroplasts, the chloroplast genome of *C. japonica* var. *Sinensis* Miquel (MT554702) did not have a typical quadripartite structure with the inverted repeats (IR) region absent, and the size of *C. japonica* var. *sinensis* Miquel is131,412 bp. The overall GC content was 35.4%. The genome encoded a set of 119 genes, containing 83 protein-coding genes, 32 tRNA genes, and 4 rRNA genes. To confirm the phylogeny of *C. japonica* var. *Sinensis* Miquel, 12 complete genomes were obtained from GenBank, and were aligned using HomBlocks software (Bi et al. 2018). A maximum likelihood (ML) tree was inferred using RAXML version 8.2.9 (Stamatakis 2014), with the combined rapid bootstrap (1000 replicates) and search for ML tree (the ‘-f a’ option).The result of ML phylogenetic tree showed that *C. japonica* var. *sinensis* Miquel is closely related to *C. japonica* (Thunb. ex L. f.) D. Don (Figure 1). The chloroplast genome of *C. japonica* var. *sinensis* Miquel will provide useful genetic information for further study on genetic diversity and conservation of Cupressaceae species.

**Figure 1.**
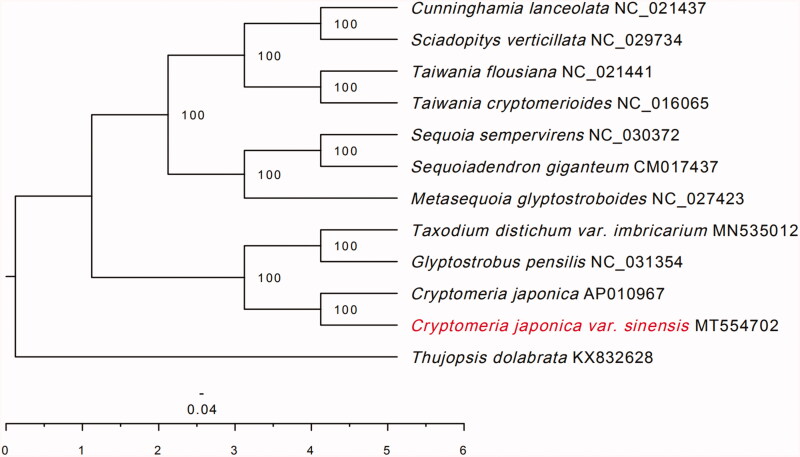
The maximum-likelihood (ML) phylogenetic tree based on the 12 plant chloroplast genome. Values along branches correspond to ML bootstrap percentages.

## Data Availability

The data that support the findings of this study are openly available in GenBank at https://www.ncbi.nlm.nih.gov/nuccore/MT554702/, reference number MT554702.
